# Draft Genome Sequence of *Paenibacillus* sp. Strain DMB20, Isolated from Alang Ship-Breaking Yard, Which Harbors Genes for Xenobiotic Degradation

**DOI:** 10.1128/genomeA.00554-15

**Published:** 2015-06-11

**Authors:** Binal Shah, Kunal Jain, Namrata Patel, Ramesh Pandit, Anand Patel, Chaitanya G. Joshi, Datta Madamwar

**Affiliations:** a Environmental Genomics and Proteomics Lab, BRD School of Biosciences, Satellite Campus, Sardar Patel University, Bakrol, Gujarat, India; b Department of Animal Biotechnology, College of Veterinary Science and Animal Husbandry, Anand Agricultural University, Anand, Gujarat, India

## Abstract

*Paenibacillus* sp. strain DMB20, in cometabolism with other *Proteobacteria* and *Firmicutes*, exhibits azoreduction of textile dyes. Here, we report the draft genome sequence of this bacterium, consisting of 6,647,181 bp with 7,668 coding sequences (CDSs). The data presented highlight multiple sets of functional genes associated with xenobiotic compound degradation.

## GENOME ANNOUNCEMENT

With the intensity at which industrialization has increased, it is impossible to deny that the environment has changed. Over the years, it has been recognized that the most important and perhaps the only chance of counteracting the devastating consequences of xenobiotic compounds on the environment lies largely in the unexplored genetic pool of the microbial world. Since bacteria have evolved for >3 billion years, their exposure to a wide variety of xenobiotic compounds has created the selective pressure necessary for an evaluation of catabolic enzymes and pathways capable of modifying or degrading unpalatable recalcitrant compounds. Thus, we have characterized the genome of *Paenibacillus* sp. strain DMB20 from one such environment perturbed with anthropogenic activities, the Alang ship-breaking yard, Bhavnagar, Gujarat, India. DMB20 is one of the strains of a consortium capable of reducing and degrading azo compounds. The genome sequence of *Paenibacillus* sp. DMB20 will be useful because of the wealth of its molecular clues in the remediation of xenobiotic compounds.

Whole-genome shotgun sequencing of the DMB20 genome was performed using the 318 chip and 400-bp chemistry on an Ion Torrent PGM platform. The draft genome of *Paenibacillus* sp. DMB20 was *de novo* assembled using the CLC Genomics Workbench software (CLC bio-Qiagen, Aarhus, Denmark), MIRA (1), and GS *de novo* assembler version 2.6. Contigs >500 bp from all three assemblies were reassembled in CISA (2). The assembly by CISA resulted in 58 contigs with sizes from 1,183 to 558,283 bp. The draft genome of *Paenibacillus* sp. DMB20 consists of 6,647,181 bp, with a 50.24% G+C content. Gene annotation and screening for RNAs were performed using the Rapid Annotations using Subsystems Technology (RAST) server (3). Genome annotation revealed 7,668 coding sequences (CDSs), 77 tRNA loci, and 9 rRNA genes.

In the *Paenibacillus* sp. DMB20 genome, 664 genes were annotated for carbohydrate metabolism, 440 for amino acids and its derivatives, and 115 for the stress response (including 18 for detoxification and 27 for nonsubcategorized stress). The genes for nonsubcategorized stress response include 7 flavohemoglobin genes. Flavohemoglobins (flavoHbs) are widely distributed among bacteria and yeasts as flavin adenine dinucleotide (FAD)/NAD-dependent reductases and oxidases. The functional annotations of flavoHbs are still contended, but various physiological roles linked to cellular responses for oxidative and nitrosative stress have been proposed (4). The presence of these genes in *Paenibacillus* sp. DMB20 confirms the role of the organism in the degradation of azo compounds and their intermediates, which are mostly driven by FAD/NAD-dependent oxidoreductases (5, 6). In addition, the DMB20 genome harbors genes for the metabolism of aromatic compounds, selenate and selenite uptake, and antibiotic and heavy metal resistance. Annotation for heavy metal resistance is endorsed by the isolation of the organism from a metal-contaminated site (7, 8).

In-depth analysis of the *Paenibacillus* sp. DMB20 genome is a prerequisite for understanding its role in the metabolism of xenobiotic compounds.

### Nucleotide sequence accession numbers. 

These whole-genome shotgun project data for *Paenibacillus* sp. DMB20 have been deposited at DDBJ/EMBL/GenBank under the accession no. LAZU00000000. The version described in this paper is version LAZU01000000.
